# Effect of Different Silicon Sources on Yield and Silicon Uptake of Rice Grown under Varying Phosphorus Rates

**DOI:** 10.3390/plants6030035

**Published:** 2017-08-29

**Authors:** Flavia B. Agostinho, Brenda S. Tubana, Murilo S. Martins, Lawrence E. Datnoff

**Affiliations:** 1 School of Plant, Environmental, and Soil Sciences, Louisiana State University AgCenter, Baton Rouge, LA 70803, USA; fagost2@lsu.edu (F.B.A.); MMartins@agcenter.lsu.edu (M.S.M.); 2 CAPES Foundation—Ministry of Education of Brazil, Brasilia 70040-031, Brazil; 3 Department of Plant Pathology & Crop Physiology, Louisiana State University AgCenter, Baton Rouge, LA 70803, USA; LDatnoff@agcenter.lsu.edu

**Keywords:** foliar Si solution, Si deposition, Si uptake, silicon, phosphorus, arsenic, rice, yield

## Abstract

A series of pot experiments were conducted to: (1) evaluate the effects of different Si sources (soil- and foliar-applied) on grain yield and Si accumulation of rice supplied with varying P rates, and (2) evaluate Si absorption of rice using foliar- and soil-applied Si fertilizers. Three P rates, (0, 112, and 224 kg ha^−1^) combined with five Si treatments (wollastonite and slag applied at 4.5 ton ha^−1^ and one foliar Si solution applied at 20, 40 and 80 mg Si L^−1^) and a check were arranged in a randomized complete block design with four replications. The presence of P and Si in the soil created a synergistic effect on soil Al, Mn, and As (*P <* 0.01), but not on rice growth and P uptake. Wollastonite and slag application were most effective in raising rice Si content than foliar applied Si (*P* < 0.001). While there was an improvement in biomass (42%) and tiller production (25%) for rice receiving foliar Si, no supporting evidence was obtained in these experiments to verify leaf surface Si absorption. The application of Si-rich materials to soil still remains the most effective method for enhancing Si uptake by plants.

## 1. Introduction

Rice (*Oryza sativa*) is a staple food crop that accounts for more than 22% of world’s population calorie intake, with Asia and Africa as the largest consuming regions [1]. For the third consecutive year, rice consumption was reported to exceed production, and ending stocks in 2015/2016 are expected to decline 15% from a year earlier, the lowest global ending stocks since 2007/2008 [2]. Climate changes such as extreme weather, unexpected temperature and rainfall fluctuations have affected crop productivity [3,4]. Abdullah [5] reported that a 1 °C increase in daily average temperature in the peninsular nation of Malaysia might reduce rice yield by 10%. In addition, according to Tao et al. [6], rice yield reduction would range from 6% to 19%, 14% to 32% and 24% to 40% for global mean temperature increase of 1, 2 and 3 °C, respectively. Other negative effects were also noted for atmospheric carbon dioxide (CO_2_) concentration of 400–800 ppm and precipitation fluctuations of ±14% [7].

An effective soil nutrient management is an essential component of crop production, responsible for increasing and sustaining crop yield at high levels [8]. All plant-essential nutrients already have established fertilization programs for rice, except the micronutrients chloride (Cl), manganese (Mn), molybdenum (Mo), and nickel (Ni), that might be supplied through impurities or composition of common-applied fertilizers [9]. Interestingly, the only non-essential nutrient that is included in the guidelines for rice fertilization is silicon (Si) [9]. Silicon plays an important role in the mineral nutrition of plants, especially for the high accumulator species, such as rice. Its benefits include enhancing plant defense response against diseases [10], protecting plants against insect pests [11], increasing plant photosynthesis and growth [12], preventing lodging [13], alleviating water [14] and mineral toxicity stresses [15,16], and improving fertilizer use efficiency [17].

Studies have indicated that Si fertilization enhances plant phosphorus (P) utilization by increasing both P content of rice [18] and phosphate fertilizer efficiency [19]. Whereas rice shoots from plants cultivated in Si solutions had twice the inorganic P content compared to shoots without Si treatment [20], superphosphate application along with Si fertilizer-enhanced rice P absorption by 8% [19]. The effect of Si on P availability was initially thought to be related to Si influences on soil pH, when it was applied as calcium (Ca) and sodium (Na) silicate [21,22,23]. Later, the chemical similarity between phosphate (H_2_PO_4_^−^) and silicate (H_3_SiO_4_^−^) ions was believed to govern this interaction [24]. There is a strong competition between H_2_PO_4_^−^ and H_3_SiO_4_^−^ ions for specific soil sorption sites [24], in which previously adsorbed H_2_PO_4_^−^ is displaced by H_3_SiO_4_^−^ and then became available for plant uptake [25,26,27,28,29,30].

Low Si content was reported to be associated with geologically old soils [31]. Moreover, it is common to find depletion of plant-available Si in soils where rice is cultivated for a long time [16]. In some countries, such as Japan, the practice of Si fertilization is already common in rice fields [32]. Silicon is commonly applied to soil as slags from iron (Fe), ferronickel, and manganese (Mn) ore smelters [33]. Slags are abundant and an inexpensive Si source, but require application at high rates [34,35]. The foliar application of Si-containing solution was proposed as an alternative Si fertilization method [36]. A number of research findings have demonstrated the positive effect of foliarly applied Si in suppressing a foliar plant disease in different crops, such as rice [37], wheat (*Triticum aestivum*) [36], grape (*Vitis vinifera*) [38], cucumber (*Cucumis sativus*), zucchini (*Cucurbita pepo*), and muskmelon (*Cucumis melo*) [39]. The suppressive effects are attributed to the deposition of dried Si solution affecting pathogen infection via ions, or a change in pH on the leaf surface [10,38,39,40]. On the other hand, there are other reports suggesting that the Si plant content increased by foliar application of Si [36,41,42]. These are interesting findings, especially since no functional Si transporter genes have been reported for leaves to date.

Silicon uptake through rice roots is mediated by specific transporters [43,44]. These transporters were characterized and identified as low-Si genes (Lsi1 and Lsi2), and are responsible for transporting Si from the soil solution to the root cells (influx, Lsi1) and from inside to the outside of the root cells (efflux, Lsi2) [44,45]. Recently, three Si transporters (Lsi2, Lsi3 and Lsi6) were also identified in the rice node [46]. While Si transporters in the roots facilitate uptake of the element by the plant, Si transporters in the node are involved in intervascular transfer, which is required for the preferential distribution of Si to the leaves and grains [46]. This study was conducted to: (1) evaluate the effects of different Si sources (soil- and foliar-applied) on grain yield and Si accumulation of rice supplied with varying P rates, and (2) evaluate Si absorption of rice using foliar- and soil-applied Si fertilizers.

## 2. Results

### 2.1. Effect of Silicon and Phosphorus on Rice Agronomics, Phosphorus, and Silicon Uptake

There was no significant Si and P interaction effect on measured plant variables across all growth stages and at harvest (Table 1). Similarly, plant variables did not respond to P rate treatment. On the other hand, a significant effect of Si treatment was observed for measured plant variables. At booting stage, plants which received 40 mg L^−1^ of foliar Si solution tended to have higher biomass yield (36%) and tiller count (12.5%) than wollastonite-treated plants (actual *P*-values 0.094 and 0.060, respectively). These responses were not carried over to flowering stage or at harvest. At flowering, the solution application of 20 and 80 mg Si L^−1^ enhanced rice biomass by 42% compared to wollastonite. At this stage, plants applied with Si solution at 80 mg L^−1^ also produced on average two tillers more than plants applied with wollastonite (Table 1). At harvest, no difference for tiller count was observed across Si treatments (Table 1). In reference to the check, the application of wollastonite and Si solution (across rates) did not result in higher biomass and grain yield (Table 1). Slag-treated plants had significantly lower biomass and grain yield than plants applied with 80 mg L^−1^ foliar Si.

**Figure 1:**
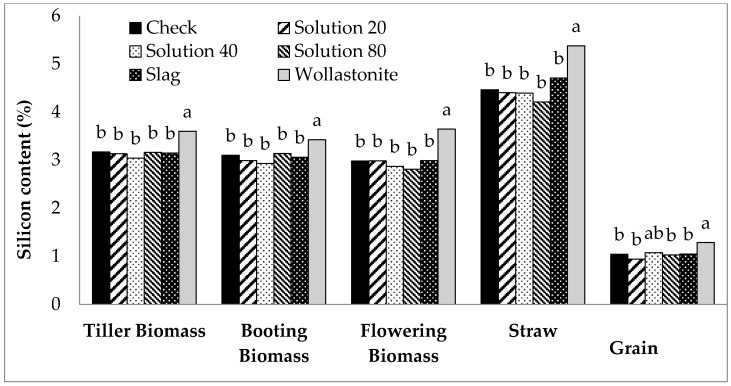
Effect of silicon treatments across phosphorus rates on silicon content of rice biomass at tiller, booting, and flowering, and of straw and grain at harvest. Bars labeled with the same letter within sampling time are not significantly different at *P* < 0.05 according to Tukey’s test. Slag and wollastonite rates: 690 and 1190 kg Si ha^−1^, respectively.

**Figure 2:**
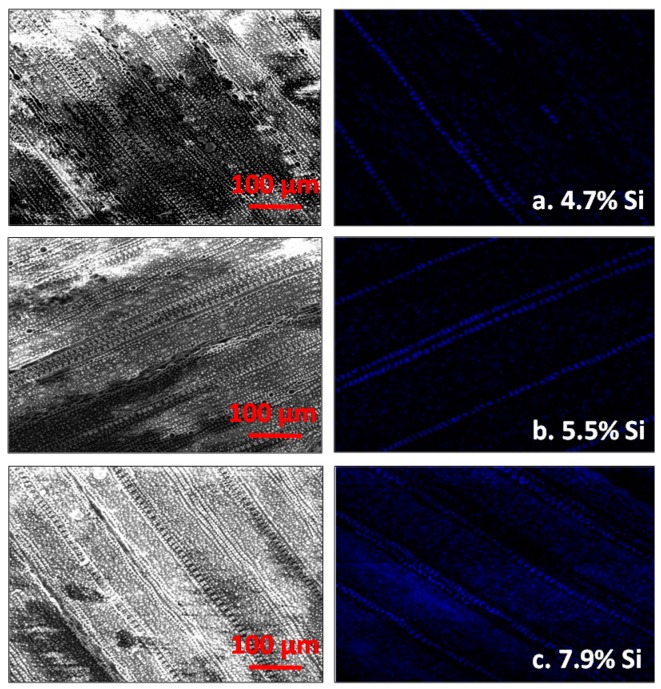
Scanning electron microscope image (400 times magnification) and energy dispersive X-ray (EDX) silicon (blue) mapping of adaxial leaf surface at flowering for check (**a**), foliarly applied silicon at 40 mg L^−1^ (**b**), and wollastonite (**c**). Red bar = 100 µm.

**Figure 3:**
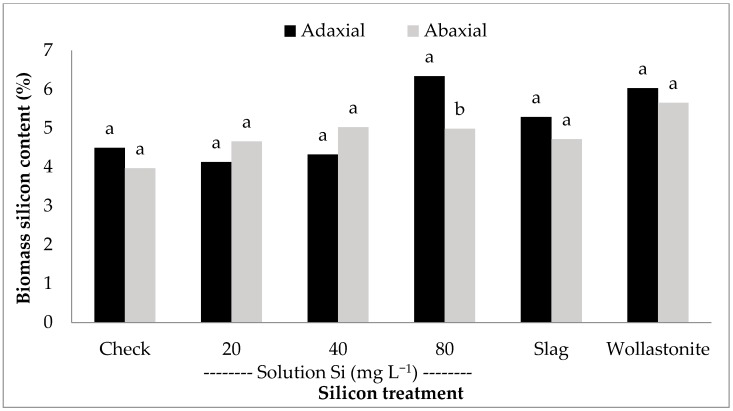
Silicon content of rice adaxial and abaxial leaf surface under scanning electron microscopy (SEM) and EDX with different silicon treatments. Bars labeled with the same letter within Si treatment are not significantly different at *P* < 0.05 according to Tukey’s test. Silicate slag and wollastonite rates: 690 and 1190 kg Si ha^−1^, respectively.

Across all growth stages and at harvest, wollastonite application consistently increased the biomass Si content (*P* < 0.05, Figure 1). In reference to the check, wollastonite application increased biomass Si content at tillering, booting, and flowering stages by 12%, 10%, and 23%, respectively. This effect was carried until harvest wherein biomass Si content was increased from 4.46% (check) to 5.38% (wollastonite). Wollastonite-treated rice obtained the highest grain Si content among the treatments, except for Si solution application at 40 mg L^−1^. Scanning electron microscopy (SEM) and energy dispersive X-ray (EDX) Si mapping visually showed a greater distribution of silica bodies on the adaxial surface of rice leaves treated with wollastonite and foliar Si solution in comparison to the check (Figure 2). In general, the adaxial leaf surface tended to have higher Si content than the abaxial leaf surface for all treatments including the check; however, a significant difference was only observed for the highest rate of foliar application (80 mg Si L^−1^) (Figure 3).

**Figure 4:**
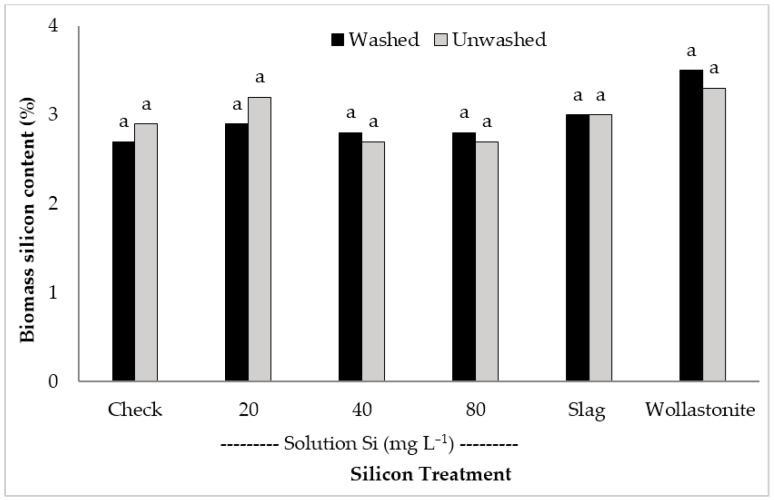
Silicon content of washed and unwashed rice biomass at flowering with different silicon treatments. Bars labeled with the same letter are not significantly different at *P* < 0.05 according to Tukey’s test. Slag and wollastonite rates: 690 and 1190 kg Si ha^−1^, respectively.

Very fine, whitish-like particles were present on the leaf surfaces sprayed with the Si solution. These particles were not observed on the controls or from plants treated via the roots with wollastonite or slag. Washing the leaves with deionised (DI) water was used to attempt to remove these fine whitish-like particles from the leaf surface so they would not interfere with the Si analysis. However, the results showed that no differences were detected between the relative Si content of washed and unwashed leaves across Si treatments (Figure 4). This result suggests that the Si leaf content was not increased by foliarly applying Si, since the percentage leaf Si content was comparable between the foliarly applied Si treatments and the check.

### 2.2. Effect of Silicon and Phosporus Fertilization on Soil pH, and Soil and Plant Nutrient Composition

**Figure 5:**
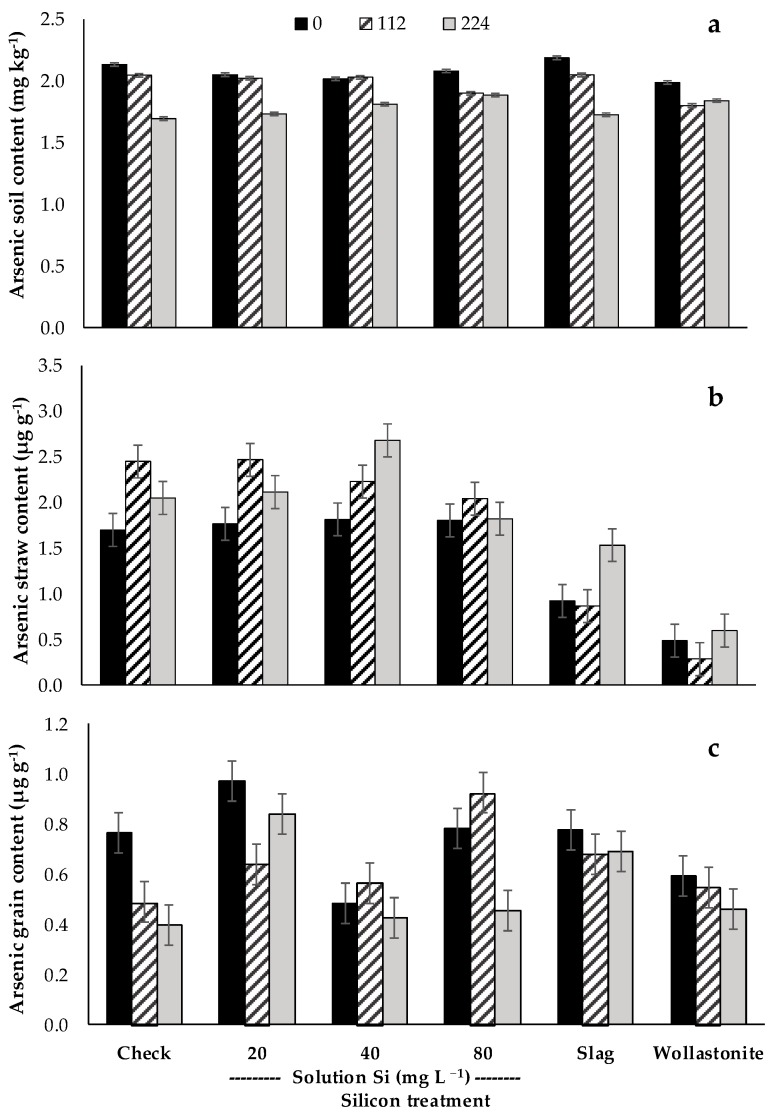
Effect of silicon treatments across phosphorus rates on arsenic content of soil (**a**), straw (**b**), and grain (**c**). Slag and wollastonite rates: 690 and 1190 kg Si ha^−1^, respectively.

The concentration of several elements in the soil and plant (straw and grain) tissue that were significantly affected by P or Si treatments or their interaction are reported in Table 2. Phosphorus application significantly increased soil P (*P* < 0.001). There was an evident reduction in soil aluminum (Al), Mn, and Fe content when P was applied (*P* < 0.01); whereas no effect on soil pH and Si content were observed. Silicon soil-sources significantly increased soil Si content compared to the check, and foliarly applied Si. Wollastonite resulted in the highest soil Si content, having 67 and 29 µg Si g^−1^ higher Si content than the check and slag treatments, respectively. In addition, there was a significant increase in soil pH with the application of slag and wollastonite (*P* < 0.05). The application of wollastonite increased soil P from 37 to 48 µg g^−1^ compared to the check (*P* < 0.05); however, slag and foliarly applied Si treatments had no observable effect on soil P content. The soil Al was reduced by wollastonite (*P* < 0.001), while slag increased soil magnesium (Mg). There was a significant P × Si interaction effect on soil arsenic (As). The analysis showed that P applied at 224 kg ha^−1^ had a greater impact in reducing soil As than 112 kg P ha^−1^ (Figure 5a). The wollastonite enhanced the reduction in soil As, regardless of whether P was applied or not, but at a lower rate (112 kg ha^−1^).

Phosphorus had no effect on straw and grain P content, but significantly reduced the Mn and Fe content in straw and grain, respectively (*P* < 0.05). Plants which received 224 kg P ha^−1^ reduced Mn straw content from 417 to 358 mg kg^−1^ (check) (*P* < 0.01), whereas there was 33 mg kg^−1^ less Fe content in rice grain receiving 224 kg P ha^−1^, in comparison to the check (*P* < 0.05). Phosphorus rate had no effect on Si content of plants, and neither did the Si treatments on plant P content (Table 2). There was a significant reduction in Mn content in straw and grains of plants treated with wollastonite (*P* < 0.001). An increase in Mg content of straw was also observed for slag-treated rice (*P* < 0.001). In general, the As content of rice straw increased when P was applied (Figure 5b). The foliarly applied Si had no apparent effect on the As content of rice straw, whereas wollastonite and slag clearly reduced As content, on average, by about 100%. Between the check and wollastonite treatments, rice treated with increasing P showed a reduction in As content of the grain (Figure 5c). Without P, wollastonite significantly reduced As in grain, whereas in the presence of P (112 and 224 kg ha^−1^) grain As content of both check and wollastonite treatment were similar. There was no clear effect of both foliar Si and P on As content of rice grain, whereas the combined application of silicate slag and P application exacerbated grain quality by raising As content.

### 2.3. Evaluation of Silicon Absorption of Rice Treated with Foliar- and Soil-Applied Silicon Fertilizers

**Figure 6:**
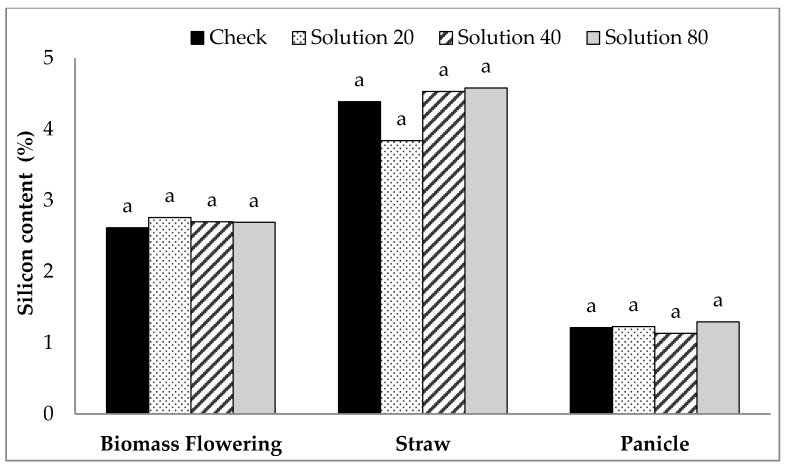
Effect of foliarly applied silicon at different rates on silicon content of biomass at flowering and straw and panicle at harvest. Bars labeled with the same letter within sampling type are not significantly different at *P* < 0.05 according to Tukey’s test.

Foliarly applying Si to the whole rice plant versus only the leaves from the third primary tiller of rice had no final effect on dry biomass and plant Si content in comparison to the control (*P* > 0.05). Analysis of variance (ANOVA) revealed that no effect was observed from different rates of foliarly-applied Si on rice biomass production and yield (data not shown). No increase in plant Si content was observed for both straw and panicles (Figure 6). Moreover, the Si content of biomass, straw and panicle from tillers which received foliarly applied Si was the same as the control tillers (Table 3).

**Figure 7:**
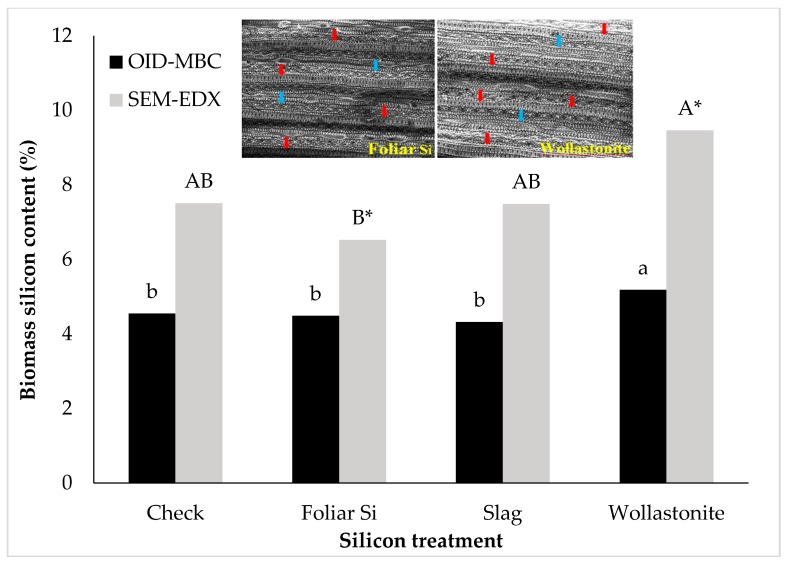
Effect of silicon treatments on leaf silicon content based on Oven Induced Digestion–Molybdenum Blue Colorimetric (OID-MBC) and Scanning Electron Microscopy–Energy Dispersive X-ray (SEM-EDX). Bars labeled with the same upper or lower case letters are not significantly different at *P* < 0.05 according to Tukey’s test. Inset (*): Scanning electron microscopy images (400 times magnification) of rice leaves showing silica bodies on leaf surface of rice treated with foliarly applied Si (1) and wollastonite (2). Red arrows = dumbbell shape silica bodies; blue arrows = globular shape silica bodies.

Leaves of rice washed with DI water and 2% nitric acid (HNO_3_) acid were analyzed for Si content using the Oven-Induced-Digestion (OID)—Molybdenum Blue Colorimetric (MBC) procedure and SEM-EDX analysis. No difference was observed in Si content of leaves washed with DI water and with 2% HNO_3_ (Table 4). The leaf Si content of rice treated with wollastonite was higher compared to foliarly applied Si and slag-treated rice plants (Figure 7). Based on the SEM-EDX analysis, biomass Si content of wollastonite-treated rice was higher than the foliarly-Si treated rice. The SEM image showed greater number of silica bodies in leaf surface under wollastonite treatment compared to foliarly applied Si treatment (Figure 7). This outcome was consistent using the OID-MBC procedure. The samples taken from the water and 2% HNO_3_ acid which were used to wash the leaves were also analyzed for Si content. There were differences in Si content in the washing solution (DI and 2% HNO_3_) where 2% HNO_3_ acid consistently had higher Si content than the DI water across the Si sources including the check (Table 4). Numerically, the amount of Si that was washed from the leave surfaces treated foliarly with Si was higher than those treated with slag or wollastonite.

## 3. Discussion

While P application increased soil P level, there was no impact observed on the agronomics of rice. On the other hand, both agronomics and Si uptake of rice responded to Si treatments. The significant effect of Si sources on tiller count and biomass was found to be more pronounced between foliarly applied Si and wollastonite treatments, as opposed to the Si sources versus the check. In contrast, Prakash et al. [47] observed an increased number of tillers in comparison to the check in a wetland rice field trial using foliarly applied silicic acid at 2 and 4 mL L^−1^. In agreement with our findings, results from other studies using foliarly applied Si showed no significant increase in the growth of plants cultivated under greenhouse conditions [36,48]. Researchers have documented the benefits of foliarly applied Si only when plants are under stress [36]. For example, Guével et al. [36] observed that plant growth did not improve with foliar applications of two different Si products in comparison to the check under greenhouse conditions, except when the plants were infected by powdery mildew. The environment under greenhouse conditions is usually controlled, and plants are usually exposed to minimal or no stress [35]. This finding might partially explain the lack of agronomic responses of rice to Si in the present study. Deren et al. [49], Liang et al. [50], and Korndorfer et al. [51] also found no difference in yield of plants without and with Si application. On the other hand, foliarly applied Si was reported to increase the yield of rice, corn (*Zea mays*) [40], soybean (*Glycine max*), common bean (*Phaseolus vulgaris*), and peanut (*Arachis hypogaea*) [42]. However, the increased yield reported in these experiments was correlated with drought stress [41,42]. Silicon-enhanced production was associated with accumulation of total sugars and proline under this type of stress condition [52]. In the present study, rice treated with slag had lower yields in comparison to foliarly applied Si at 80 mg L^−1^. This negative effect of slag on rice yield might be attributed to its effect on soil pH [53], since incorrect pH can compromise the availability of plant-essential nutrients, which in turn will limit plant growth and yield [54].

The increased concentration of phosphate in the soil immobilized Al, Mn, and Fe; subsequently, reducing their (extractable) concentration in the soil. Moreover, the reduction of soil As with increasing P rate also demonstrated the competition between these two elements for binding sites. Phosphate is chemically analogous to arsenate, and competes for binding sites in the soil, which might also reduce arsenate availability to plants [55]. The reduction in extractable amounts of these elements resulted in reduced uptake by rice, but was not related to tiller or biomass production of rice. Both wollastonite and slag significantly raised soil Si to levels that may have caused the desorption of P, and consequently increased the P extracted in the soil based on Mehlich-3 extraction procedure. The decrease in P adsorption by Si treated-soil and the further increase in soil P content were reported by several authors [21,22,23]. While there was an increase in Si uptake with an increase in soil Si, the accompanied increase in soil P did not result in any notable increase in P uptake in the present study. The outcomes of Ma and Takahashi [20] study agreed with the results of this study, showing that the addition of Si was not accompanied by an increase in shoot P concentration. A study conducted by Lima [30] showed otherwise; Si application via soil led to a reduction in P fixation and an increase in P uptake by the plant. At a soil pH above 7, phosphates in solution start to precipitate [56]. Initially, this seemed to be the reason behind the lack of improvement in P uptake in rice treated with wollastonite and slag observed in this study. However, the precipitation of phosphate brought about by an increased in soil pH did not reduce soil P below the critical P level (35 ug g^−1^) [57]; therefore the fact that rice P uptake did not respond to P fertilization may have likely been due to sufficient supply of plant-available P.

Plants treated with Si were reported to alleviate Mn toxicity [58,59,60]. The results obtained from this study demonstrated this specific role of Si, in that the reduction in Mn content was observed for plants treated with wollastonite and slag materials. Moreover, reports were made that the high affinity of P for metals, such as Mn and Fe, might reduce its plant content, alleviating metal toxicity [61]. Because silicic acid transporters have been reported to mediate arsenite uptake in rice [62,63], high concentrations of Si might have decreased the ability of these transporters to uptake As in rice. Some evidence of this possibility was observed with wollastonite and slag. The reduction in soil As with P rate can be reflected in total As content of grain but not in the straw. Both phosphate and silicates are adsorbed by Al oxides [24]. This finding perhaps explains the observable reduction in soil Al when both soil Si and P were increased after the addition of P fertilizer and soil Si sources (slag and wollastonite).

Based on the results of two experiments, foliarly applied Si did not increase the percentage of leaf Si content in comparison to the control. This result is similar to the findings by Rezende and colleagues [64] and suggests that plant Si absorption did not take place via leaf surface absorption. Rezende et al. [64] observed higher amounts of Si deposits on the adaxial leaf surface in comparison to abaxial leaf surface of Si foliarly applied plants. This effect was also observed in the present study for the highest rate of foliarly applied Si (80 mg Si L^−1^). The washing of leaf samples, whether with DI water or 2% HNO_3_ acid, did not make any difference in the Si analysis of the leaf surface and biomass samples. The Si content of washing solution indicated that more Si was removed from the leaves which were applied foliarly with Si than those that received Si root applications via slag or wollastonite. The dried Si solution on the leaf surface became a thin layer of silica. This finding explains why a greater number of silica bodies were observed on the SEM image and EDX mapping of leaf surfaces collected early in the season. The dried Si bodies disappeared by harvest because of the lack of a continuous supply of foliarly applied Si. Unless applied multiple times during the growth of the plant, foliarly applied Si cannot enhance the formation of silica gels on the surface of new growing leaves. However, a positive response to foliarly applied Si has been reported, but was not directly attributed to a percentage increase in foliar Si content. For example, Sousa et al. [65] observed an increase in the yield of corn foliarly applied with potassium silicate; however, the results were attributed to the joint effect of Si and K in the plant, not Si alone. Although the Si solution used in this study contained a carrier and low concentrations of other nutrients besides Si, no effect on rice nutrient uptake was observed based on the elemental composition analysis of plant tissue samples. Another positive response to foliarly applied Si was observed by Crusciol et al. [41], wherein the rice flag leaf Si content for foliarly applied Si was greater than the non-treated control. However, in this study, the increased in Si content from foliarly applied Si under field conditions might have resulted from Si solution runoff into the soil, and subsequent uptake by the roots. Drenching soil with Si is another application method. A significant increase in biomass Si content was detected when liquid Si sources were applied to soil or soil-less media [66,67]. Kanto et al. [67] reported that Si content in strawberry (*Fragaria* L.) was increased by 30% when a liquid Si source was applied to the plots as a soil drench. In a hydroponic study, the addition of liquid potassium silicate to a nutrient solution increased the amount of Si uptake by the plant [66].

The relationship between P and Si was not clearly demonstrated in the present study. Moreover, there was no clear evidence that proved the absorption of Si through the rice leaf surface. Silicon applied via soil was consistently more effective than foliarly-applied Si in enhancing the Si content of rice. For future studies, it is essential to evaluate several types (e.g. different carrier, pH, ionic vs. complexed form) of Si solutions. Perhaps the unique chemical and physical properties of nanotechnology could help with this absorption issue.

## 4. Materials and Methods

### 4.1. Treatment Structure and Experimental Design

Bulk soil samples were collected in a rice field in Eunice (Evangeline Parish), Louisiana. The soil was Crowley-Vidrine complex with a silt-loam to silt-clay texture, and poor drainage (SSURGO-USDA, 2015), slightly acidic (6.14, 1:1 pH in water), with low Mehlich-3 soil test P, and low Si levels based on a 0.5 M acetic acid extraction procedure (Table 5). The treatment structure for the Si × P experiment was a two-way factorial with five Si treatments and three P rates with four replications arranged in a randomized complete block design. Phosphorus rates were 0, 112 and 224 kg of P ha^−1^ applied as triple superphosphate (TSP, 46% P, Magnolia, MS, USA), and the Si treatments included Si soil sources wollastonite (Vansil^®^, 23% Si, Norwalk, CT, USA) and slag (Plant Tuff^®^, 14% Si, Dearborn, MI, USA) applied at 4.5 ton ha^−1^, and a Si solution (6000 mg Si L^−1^) source applied at 2, 4 and 8 L ha^−1^, diluted to a final foliar-application volume of 600 L ha^−1^. A check Si (no Si application) was also included for each P rate. Both wollastonite and slag were applied only once before planting, while solution Si were applied at early tillering, booting, and early flowering stages. The solution Si delivered 12, 24, and 48 g of Si ha^−1^ at 2, 4, and 8 L ha^−1^, respectively, while wollastonite and slag application delivered 1190 and 690 kg of Si ha^−1^, respectively.

Two separate experiments were also conducted to examine solution Si absorption in rice leaves. In the first experiment, the same Si solution was used but applied at rates of 0 (deionized water, DI), 20, 40, and 80 mg of Si L^−1^, diluted to a final volume of application of 600 L ha^−1^. The solution was sprayed either to whole plants, or to leaves of the primary third tiller of each plant. The application was done three times: at early tillering, booting, and early flowering stages. For the second experiment, three Si sources including a check were tested. The treatments were (1) foliar-applied Si solution at 80 mg of Si L^−1^, (2) soil-applied slag at 4.5 ton ha^−1^, (3) soil-applied wollastonite at 4.5 ton ha^−1^, and (4) a check treatment accomplished by foliar application of DI water. For this experiment, the Si solution was applied only once at early tillering stage, and strictly to the adaxial (upper) leaf surface.

### 4.2. Experiment Establishment

Plastic pots with 13 L capacity were filled with 11 kg of air-dried and sieved soil. All pots received 90 kg ha^−1^ K (KCl, 60% K) and 6 kg ha^−1^ Zn (ZnSO_4_, 23% Zn) before planting. Both P and dry Si source (wollastonite and slag) treatments were established before sowing by spreading and incorporating the fertilizers into the soil. Ten seeds of rice variety CL151 were sown per pot and, at four-leaf growth stage, seedlings were thinned to six plants per pot. The first N fertilization (urea-45% N) was applied right after sowing at 115 kg ha^−1^. Two weeks after sowing, pots were flooded and a 2.5 cm-water column was maintained until 2–3 weeks before harvesting. Additional N and K fertilizer were applied five and 20 days after flooding at 56 and 68 kg ha^−1^, respectively. Silicon solution was foliarly applied using a pressurized handheld sprayer (Stihl^®^ SG 10, Virginia Beach, VA, USA). During the application, the base of the plants was covered with a plastic sheet to prevent run-off of Si solution into the soil. The same crop establishment and management were applied to all experiments.

### 4.3. Biomass Sampling, Harvesting, and Soil Sampling

For both the Si × P and first experiments, biomass sampling was done one week after each solution Si application. Two plants per pot were selected for each sampling. Tiller number was recorded and separated into two groups: one was washed thoroughly with DI water before oven-drying and the other group was left unwashed. Washing biomass samples prior to analysis was done to remove Si solution that may have dried on the surface of sprayed leaves and stems. At harvest, panicles were separated from tillers, and their numbers were noted before placing them into separated bags. Rice grains were detached from panicle by hand and weighed. Biomass and harvest samples were oven-dried at 65 °C for 72 h, weighed, ground, and analyzed for Si and elemental composition. For the second experiment, plants were harvested one week after foliar application, and separated into two groups: one was washed with DI water and other with 2% HNO_3_ before oven-drying. Washing was done in batches of 12 samples using 100 mL of either DI water or 2% HNO_3_. Samples were shaken for 2 min on a reciprocal shaker. After washing, samples of the solutions for each treatment were collected and analyzed for Si content by MBC [68]. After harvest, composite soil samples were also collected, oven-dried at 40 °C, ground, and analyzed for Si and extractable nutrient content.

### 4.4. Plant Analysis

Silicon content in plant tissue was determined by OID [69] followed by the MBC procedure [68]. For digestion, 100 mg of ground tissue sample was weighed into a 50-mL polyethylene centrifuge tubes, and 5 drops of octyl alcohol and 2 mL of hydrogen peroxide (H_2_O_2_) were added before placing it in the oven at 95 °C for 30 min. Samples were then taken out of the oven and 4 mL of 50% sodium hydroxide (NaOH) was added. Tubes were loosely capped and placed back into the oven. Every 15 min for 4 h, tubes were taken out of the oven and gently mixed using a vortex mixer. After 4 h, 1 mL of ammonium fluoride (NH_4_F) was added to the digested samples, mixed, and diluted to 50 mL with DI water. For MBC procedure, 2 mL aliquot of plant digest solution was obtained and placed into 50-mL centrifuge tube. Ten milliliters of 20% acetic acid and 2 mL of 0.26 M ammonium molybdate [(NH_4_)_6_Mo_7_O_2_] were added. Samples then were allowed to stand for 5 min before adding 2 mL of 20% tartaric acid. The sample solution was mixed again and allowed to sit for 2 min before adding 2 mL of ANSA (reducing agent composed by 0.5 mg of 1-amino-2-naphthol-4-sulphonic acid, 1.0 g of sodium sulfite and 30.0 g of sodium bisulfite). The samples were diluted with 20% acetic acid to a final volume of 30 mL, and absorbance readings were measured at 630 nm using a ultra violet (UV)-visible spectrophotometer (Hach DR 500).

For essential nutrient and heavy metal contents, plant tissue samples were digested with concentrated HNO_3_ and 30% H_2_O_2_ at 150 °C as outlined by Jones et al. [70], and analyzed using inductively coupled plasma (ICP)—Optical Emission Spectroscopy (OEM) (Spectro Arcos FH12; Kleeve, GER).

### 4.5. Soil Analysis

Silicon content was determined by 0.5 M acetic-acid extraction procedure followed by MBC [71], whereas analysis of extractable nutrient content was based on the Mehlich-3 procedure followed by ICP atomic spectrometry [72]. For soil Si content analysis, 2 g of soil was weighed into a polyethylene centrifuge tube and added with 20 mL of 0.5 M acetic acid. The tubes were shaken using a reciprocal shaker set at high speed for 1 h. Soil suspension was filtered using Whatman^®^ (Marlborough, MA, USA) No. 1 filter paper. A 0.5 mL aliquot was transferred to a centrifuge tube for MBC analysis. Ten milliliters of DI water, 0.5 mL of 1:1 HCl:water solution, and 1 mL of 10% (NH_4_)_6_Mo_7_O_2_ (adjusted for pH 7.5) were successively added to the samples. Samples were allowed to stand for 5 min before adding 1 mL of 20% tartaric acid. Samples were gently swirled for 10 s, allowed to sit for 2 min, added with 1 mL of ANSA and then with DI water to make 25 mL final volume. Absorbance reading was measured after 5 min at 630 nm using UV visible spectrophotometer.

The plant-essential nutrient and selected heavy metals contents in soil were measured by weighting 2 g of soil in a 125 mL plastic bottle, and adding 20 mL of Mehlich-3 solution (dilute acid-fluoride-EDTA solution corrected to pH 2.5). Samples were shaken for 5 min using a reciprocal shaker at high speed and filtered using Whatman^®^ filter paper No. 42. Clear filtrates were transferred to 10-mL plastic tubes and analyzed by ICP atomic spectrometry.

### 4.6. Scanning Electron Microscopy and Energy Dispersive X-ray Analysis

Scanning electron microscopy coupled to EDX microanalysis mapping was used to determine relative Si content on leaf surface, and silica deposition in the adaxial and abaxial leaf surface of rice. Before drying the leaf samples, small sections were cut and stored in the refrigerator for SEM-EDX analysis. Under SEM, the magnification of samples’ images was set to 400 times magnification, and system operation at voltage of 20 kV. This technology relies on atomic excitation by electron beams, which provides a semi-quantitative determination of nutrient content by proportionality of scanned area [73]. Silicon peaks were proportionally quantified according to leaf carbon (C), oxygen (O), chlorine (Cl) and K contents. Three readings per sample were taken to increase data reliability.

### 4.7. Statistical Analysis

The data were subjected to ANOVA using SAS 9.4 (SAS Institute, Cary, NC, USA, 2012). Using PROC MIXED, P rates, Si source, and their interaction were assigned as fixed effects, whereas replication was set as a random effect. Treatment means were compared using the Tukey test at *P* < 0.05.

## 5. Conclusions

Rice agronomic parameters were not affected by P rates, but a significant effect of P on soil nutrient composition, as well as on plant nutrient uptake was observed. Foliar application of Si solution resulted in higher biomass and tiller count than the soil-applied wollastonite, but only during the early growth stage of rice. Silicon applied to roots via soil (wollastonite and slag) and leaves (Si solution) did not result in a significant increase in rice P content in straw and grain, and neither did the P rates affect Si plant content. However, a corresponding increase in soil P content (from 37 to 48 µg g^−1^) with wollastonite application suggested that these two nutrients (P and Si) may have similar soil binding sites. Increased concentration of phosphate in the soil immobilized Al, Mn, Fe, and As, subsequently reducing their concentration in the soil. Silicon application (wollastonite) enhanced the reduction of soil As, regardless of whether P was applied or not, but at lower rate. These results suggest that P, Si and As compete for the same binding sites in the soil, but that P has greater effect on As content than Si. Moreover, without P, wollastonite significantly reduced As levels in grain.

There was no clear evidence collected from this series of greenhouse studies that confirmed the absorption of Si via leaf surface. Foliarly applied Si did not increase Si content and uptake by rice. Unless applied multiple times over the different plant growth stages, foliarly applied Si cannot enhance silica gel formation on the surface of new growing leaves. Silicon applied to the roots via soil was consistently more effective than foliarly applied Si in enhancing Si content of plants, but this result depended on the Si source (wollastonite or slag) and the rate of application. Absorption of Si through the roots appears to be the only mechanism thus far by which Si can be taken up by plants. The liming potential of soil Si source should be taken into consideration when making recommendations especially for high-pH or basic soils. The value of raising soil Si may be outweighed by the negative impact of increasing soil pH to a level that can compromise availability and solubility of other plant-essential nutrients. However, where liming is needed, Si sources could be used as an alternative method for raising soil pH, and at the same time increasing Si content. Silicon sources have similar Ca carbonate equivalents for raising soil pH and would save application costs for lime.

## Figures and Tables

**Table 1:** Effect of phosphorus rate and silicon treatments on rice tiller count and biomass at booting, flowering, and harvest.

Treatments	Booting		Flowering		Harvest		
	Tiller	Biomass	Tiller	Biomass	Tiller	Biomass	Grain
	count pot^−1^	g pot^−1^	count pot^−1^	g pot^−1^	count pot^−1^	g pot^−1^	g pot^−1^
P rate (P), kg ha^−1^							
0	9.3	17.3	6.3	19.1	17.1	73.9	42.8
112	8.5	17.2	5.8	18.2	17.1	71.6	41.6
224	8.8	19.0	6.3	19.3	17.0	73.0	42.4
*P*-value	NS	NS	NS	NS	NS	NS	NS
Si treatment (Si)							
Check	7.7 e	17.2 ab	6.0 ab	17.5 ab	17.4	74.6 ab	43.2 ab
Solution, 20 mg L^−1^	9.1 bc	19.5 ab	6.7 ab	21.0 a	16.8	73.6 ab	42.8 ab
Solution, 40 mg L^−1^	9.9 a	20.9 a	5.9 ab	19.0 ab	17.6	74.6 ab	43.6 ab
Solution, 80 mg L^−^^1^	8.3 de	16.9 ab	7.0 a	21.0 a	18.1	79.4 a	45.8 a
Slag	9.4 ab	17.3 ab	5.9 ab	19.0 ab	16.3	64.6 b	37.2 b
Wollastonite	8.8 cd	15.3 b	5.3 b	14.8 b	16.1	70.2 ab	40.7 ab
*P*-value	NS	NS	<0.05	<0.05	NS	<0.05	<0.05
P × Si	NS	NS	NS	NS	NS	NS	NS

Note: Means followed by the same letter within columns are not significantly different according to Tukey’s test.

**Table 2:** Effect of phosphorus and silicon treatments on soil pH, and soil and plant nutrient composition.

Treatments		Soil						Straw				Grains			
	pH	Si	P	Al	Mn	Fe	As	P	Mg	Mn	As	P	Fe	Mn	As
		mg kg^−1^						%		mg kg^−1^		%	mg kg^−1^		
P rate (P), kg ha^−1^															
0	7.33	57	24 c	643 a	149 a	905 a	0.424	0.049	0.086	417 a	1.415	0.254	147 a	36.9	0.728
112	7.35	53	39 b	605 b	134 b	877 ab	0.399	0.048	0.087	416 a	1.723	0.244	122 ab	36.3	0.640
224	7.28	58	59 a	575 b	130 b	847 b	0.385	0.050	0.081	358 b	1.799	0.250	114 b	35.5	0.544
*P*-value	NS	NS	<0.001	<0.001	<0.001	<0.01		NS	NS	<0.01		NS	<0.05	NS	
Si treatment (Si)															
Check	6.97 b	39 c	37 b	651 a	140	894	0.398	0.052	0.074 bc	423 a	2.066	0.234	121	38 a	0.050
Solution, 20 mg L^−1^	7.01 b	36 c	38 ab	634 a	126	860	0.399	0.052	0.089 b	442 a	2.116	0.263	128	38 a	0.817
Solution, 40 mg L^−^^1^	7.13 b	37 c	38 ab	637 a	133	903	0.406	0.050	0.079 bc	403 a	2.242	0.263	106	40 a	0.491
Solution, 80 mg L^−^^1^	7.08 b	37 c	45 ab	631 a	142	873	0.411	0.053	0.082 bc	426 a	1.889	0.239	126	40 a	0.720
Slag	7.79 a	77 b	39 ab	635 a	143	877	0.433	0.046	0.113 a	393 a	1.106	0.250	139	31 b	0.715
Wollastonite	7.94 a	106 a	48 ab	460 b	140	851	0.370	0.042	0.070 c	300 b	0.455	0.250	148	31 b	0.532
*P*-value	<0.05	<0.001	<0.05	<0.001	NS	NS		NS	<0.001	<0.001		NS	NS	<0.001	
P × Si	NS	NS	NS	NS	NS	NS	<0.01	NS	NS	NS	<0.05	NS	NS	NS	<0.01

Note: Means followed by the same lowercase letter within columns for each factor (i.e., P, Si) are not significantly different according to Tukey’s test. Slag and wollastonite rates: 690 and 1190 kg Si ha^−1^, respectively.

**Table 3:** Silicon content of biomass and panicle of tillers with and without foliarly applied silicon.

Treatment	Biomass Si at Flowering (%)	Straw Si (%)	Panicle Si (%)
Tiller with foliar Si	2.97	4.27	1.17
Tiller without foliar Si	2.92	4.57	1.16
*P*-value	NS	NS	NS

Note: NS = non-significant. Means are not significantly different at *P* < 0.05 according to Tukey’s test.

**Table 4:** Effect of washing the leaves with deionised (DI) water and 2% HNO_3_ on silicon content of rice leaves, and silicon content of DI and 2% HNO_3_ washing solution under different silicon treatments.

Variable	Washing Solution	Silicon treatments			
		Check	Foliar Si	Slag	Wollastonite
Silicon in plant biomass (%)	DI water	4.55	4.48	4.67	5.09
	2% HNO_3_	4.68	4.29	3.62	5.02
*P*-value		NS	NS	NS	NS
Silicon in washing solution (µg mL^−1^)	DI water	0.01 b	1.14 b	ND	0.17 b
	2% HNO_3_	0.16 a	1.63 a	0.85	1.28 a
*P*-value		<0.05	<0.05		<0.05

Notes: NS = non-significant, ND = not detected. Means followed by the same letter within columns are not significantly different according to Tukey’s test.

**Table 5:** Soil characterization analysis before experiment establishment.

Texture	pH (1:1 Water)	Sum of Bases (cmol_c_ dm^−3^)	CEC (cmol_c_ kg^−1^)	Extractable Nutrients (mg kg^−1^)								
				Si	P	K	Ca	Mg	Na	S	Cu	Zn
Silt Loam	6.14	5	12	37	16	39	756	112	20	14	1	2
